# Comparing Methods to Determine Complete Response to Chemoradiation in Patients with Locally Advanced Cervical Cancer

**DOI:** 10.3390/cancers16010198

**Published:** 2023-12-31

**Authors:** Kim van Kol, Renée Ebisch, Maaike Beugeling, Jeltsje Cnossen, Joost Nederend, Dennis van Hamont, Sjors Coppus, Jurgen Piek, Ruud Bekkers

**Affiliations:** 1 Department of Obstetrics and Gynecology, Catharina Cancer Institute, Catharina Hospital, 5623 EJ Eindhoven, The Netherlandsjurgen.piek@catharinaziekenhuis.nl (J.P.); 2 Department of Obstetrics and Gynecology GROW, School for Oncology and Developmental Biology, Maastricht University Medical Center+, 6229 HX Maastricht, The Netherlands; 3 Department of Obstetrics and Gynecology, Radboud University Medical Center, 6525 GA Nijmegen, The Netherlands; renee.ebisch@radboudumc.nl; 4 Department of Radiation Oncology, Institute Verbeeten (BVI), 5042 SB Tilburg, The Netherlands; 5 Department of Radiation Oncology, Catharina Cancer Institute, Catharina Hospital, 5623 EJ Eindhoven, The Netherlands; 6 Department of Radiology, Catharina Cancer Institute, Catharina Hospital, 5623 EJ Eindhoven, The Netherlands; 7 Department of Obstetrics and Gynecology, Amphia Hospital, 4818 CK Breda, The Netherlands; dvanhamont@amphia.nl; 8 Department of Obstetrics and Gynecology, Maxima Medical Center, 5631 BM Veldhoven, The Netherlands

**Keywords:** locally advanced cervical cancer, chemoradiotherapy, MRI, 18F[FDG]-PET/CT, radiologic imaging, salvage surgery, residual disease, survival

## Abstract

**Simple Summary:**

In patients with locally advanced cervical cancer, there is still a high reported locoregional recurrence rate after chemoradiation therapy. Therefore, timely detection of locoregional residual disease is important for patients to be treated with salvage surgery. Furthermore, it is important to select only the patients who would benefit from salvage surgery and not to expose patients unnecessarily to the risks of surgery. Therefore, the aim of this study is to assess two different imaging techniques, MRI and 18F[FDG]-PET/CT, for their ability to determine the presence of locoregional residual disease after chemoradiotherapy in patients with locally advanced cervical cancer. MRI and 18F[FDG]-PET/CT were compared with pathology-proven locoregional residual disease or locoregional recurrence identified from biopsy and/or salvage surgery. The findings from this research provide insights into the ability of MRI and 18F[FDG]-PET/CT to detect locoregional residual disease.

**Abstract:**

Objectives: There is no consensus on the most reliable procedure to determine remission of cervical cancer after chemoradiotherapy (CRT). Therefore, this study aims to assess the diagnostic performance of two different imaging techniques, MRI and 18F[FDG]-PET/CT, in determining the presence of locoregional residual disease after CRT in patients with locally advanced cervical cancer. Methods: Patients diagnosed with locally advanced cervical cancer (FIGO 2009) treated with CRT were retrospectively identified from a regional cohort. The accuracy of MRI and 18F[FDG]-PET/CT in detecting locoregional residual disease was assessed with histology as the reference standard. Results: The negative predictive value (NPV) and positive predictive value (PPV) for locoregional residual disease detection of MRI and 18F[FDG]-PET/CT combined were 84.2% (95% CI 73.2–92.1), and 70.4% (95% CI 51.8–85.2), respectively. The NPV and PPV of MRI alone were 80.2% (95% CI 71.2–87.5) and 47.7% (95% CI 35.8–59.7), respectively, and values of 81.1% (95% CI 72.2–88.3) and 55.8 (95% CI 42.2–68.7), respectively, were obtained for 18F[FDG]-PET/CT alone. Conclusion: In this study, the reliability of MRI and 18F[FDG]-PET/CT in detecting locoregional residual disease was limited. Combining MRI and 18F[FDG]-PET/CT did not improve predictive values. Routine use of both MRI and 18F[FDG]-PET/CT in the follow-up after CRT should be avoided. MRI during follow-up is the advised imaging technique. Pathology confirmation of the presence of locoregional residual disease before performing salvage surgery is warranted.

## 1. Introduction

Cervical cancer is the fourth most common malignancy in women worldwide with approximately 604,127 new diagnoses in 2020 [1]. According to the 2018 Federation of Gynecology and Obstetrics (FIGO) staging system, patients with stage IIB-IVA are classified as locally advanced cervical cancer [2]. Patients with locally advanced cervical cancer are ideally treated with chemoradiotherapy (CRT), which generally consists of platinum-based chemotherapy combined with external beam radiotherapy (EBRT), followed by brachytherapy [3]. The 5-year overall survival rates after CRT range from 70% for stage IIB to 32% for stage IVA [4]. 

After treatment with CRT, recurrent disease is observed in 15–40% of cases [5,6,7,8]. These patients have an unfavorable prognosis, with a reported 5-year survival rate of less than 5% [9]. Patients treated with CRT are clinically and/or radiologically examined during the follow-up period to evaluate the response rate of CRT [10]. Detection of locoregional residual disease can be challenging due to the effects of radiotherapy [11]. Worldwide, different diagnostic procedures, such as a Magnetic Resonance Imaging (MRI), Positron Emission Tomography (18F[FDG]-PET/CT) and/or a biopsy, are used to assess remission. A meta-analysis from Sistani et al. reported the diagnostic accuracy of MRI and 18F[FDG]-PET/CT for detection of residual disease after CRT for locally advanced cervical cancer. They reported a sensitivity and specificity of 73% and 96%, respectively, for MRI and values of 86% and 95%, respectively, for 18F[FDG]-PET/CT [12]. 

When central pelvic residual disease is radiologically suspected and/or histologically proven, without signs of metastatic disease, salvage surgery can be performed [7]. Salvage surgery, such as salvage hysterectomy or exenteration, are procedures associated with a high risk of complications and a negative impact on the quality of life of patients [5,10]. Therefore, a diagnostic procedure that is highly reliable in the detection of locoregional residual disease is important to select patients who would benefit from salvage surgery. Moreover, the procedure should protect patients from unnecessary exposure to risks. Worldwide, or even nationwide, there is no consensus on the most reliable procedure to determine remission, nor the optimal time interval after CRT to perform remission procedures.

Therefore, we assessed two different imaging techniques, MRI and 18F[FDG]-PET/CT, for their ability to determine the presence of locoregional residual disease after CRT in patients with locally advanced cervical cancer. 

## 2. Methods

### 2.1. Study Design and Patient Selection

A retrospective cohort study was performed by analyzing data from the web-based registration system: Registration system Oncological Gynecology (ROGY), covering the southern region of the Netherlands. In a multidisciplinary patient consultation between hospitals in this region, information on diagnosis, treatment and follow-up is reported in the database. The southern region includes one tertiary and six secondary hospitals, and two radiation oncology centers. 

The Standards for Reporting of Diagnostic Accuracy Studies (STARD) initiative was followed [13]. All patients with cervical cancer between 2005 and 2018 were identified by two independent researchers (KvK and LA or SK). Medical records were used to supplement missing data from the ROGY database. Information on radiation dose was obtained from the Institute Verbeeten and from medical records from the included hospitals. There was no intended sample size as this was a retrospective study.

All patients diagnosed with adenocarcinoma, squamous cell carcinoma or adenosquamous carcinoma who were treated with primary CRT were included. Patients with distant metastases at the time of diagnosis, other histological subtypes, primary treatment other than CRT, and patients with incomplete follow-up were excluded. The following information from the included patients was extracted: age, FIGO 2009 stage, pathology results, dose and regimen of CRT, date of last CRT treatment, date and result of radiological imaging (MRI and/or 18F[FDG]-PET/CT), information on salvage treatment, complications, and information on locoregional recurrence of disease. Patients with missing data were excluded. 

### 2.2. Treatment

Patients were treated with EBRT 25 × 2 Gy or 28 × 1.8 Gy (five days a week) followed by 3 × 7 Gy brachytherapy or EBRT 25 × 1.8 Gy (five days a week) followed by 4 × 7 Gy brachytherapy, combined with five weekly courses of concurrent chemotherapy consisting of an intravenous infusion of cisplatin at a dose of 40 mg/m^2^ during EBRT. In the presence of pathological lymph nodes, an external boost radiotherapy was given. Complete treatment was defined as a minimum of 2 cycles of cisplatin combined with external beam radiotherapy (45–50.4 Gy) followed by brachytherapy (3–4 × 7 Gy).

### 2.3. Follow-Up

After the last CRT treatment, patients had a follow-up consultation at least every three months, with clinical examination during the first two years and then every six months during the next three years, together with laboratory testing. An MRI scan and/or 18F[FDG]-PET/CT were performed to assess the presence of locoregional residual disease according to protocol or when the practitioner considered it necessary. Therefore, some patients underwent multiple radiological imaging. In the Catharina Hospital, MRI was performed using a 3 Tesla MR system (Ingenia, Philips Healthcare, Amsterdam, The Netherlands). The imaging protocol included T2-weighted sequences of the pelvis in three directions and diffusion-weighted images (b-values until 1500 s/mm^2^) with apparent diffusion coefficient mapping. Images were obtained perpendicular to the cervix. There were no protocols available from the other included hospitals. Central assessment was performed by the expert radiologist from the multidisciplinary team, who also assessed the quality as at least sufficient. Response assessment of CRT was discussed in multidisciplinary meetings with gynecologist-oncologists, radiation oncologists, radiologists, and medical oncologists. In cases of discrepancies in the radiological examination, examination under anesthesia with biopsies was performed. Salvage surgery was performed when locoregional residual disease was radiologically suspected and/or histologically proven, without any signs of distant metastases.

### 2.4. Statistical Analysis

Descriptive statistics were used to describe patient characteristics. The sensitivity, specificity, negative predictive value, and positive predictive value were assessed for MRI scans and 18F[FDG]-PET/CT to detect locoregional residual disease. Residual locoregional disease was defined as any histologically proven and/or highly suspicious abnormality in the cervix or pelvic area at any time after CRT. Both disease detected due to scans performed to assess complete response after treatment, as well as locoregional recurrences detected during follow-up were considered the result of residual disease. If pathology results were not obtained and patients were palliatively treated because of radiologically suspected residual disease with distant metastases, it was considered as locoregional residual disease after CRT.

To assess the accuracy of both imaging techniques, the first MRI and/or 18F[FDG]-PET/CT for each patient performed after CRT were analyzed and compared with the presence or absence of locoregional residual disease. MRI and 18F[FDG]-PET/CT were considered at one time-point when both were performed within a four-week time period. As long as tumor was visible, even if there was regression compared to previous scans, it was described as locoregional residual disease. Secondly, the accuracy of all first scans was analyzed separately. Thirdly, the final radiological technique indicating presence or absence of locoregional residual disease was analyzed. Also, the time from last CRT treatment until the pathology result was obtained was analyzed. When no pathology result was available, the time from the last CRT up to the decision that pathological evaluation was not necessary because there were no signs of residual disease was taken as the endpoint. Clinical information, reference standard results and index test (MRI and/or 18F[FDG]-PET/CT) results were available to the assessors of the collected data. Complications after salvage surgery were also described. Differences were considered significant if *p* < 0.05. All the analyses were performed with the software IBM SPSS 22 (Statistical Package for Social Science). 

The study was approved by the Medical Research Ethics Committees United. The study was exempt from institutional review board approval because the data were gathered retrospectively and analyzed anonymously. 

## 3. Results

**Figure 1:**
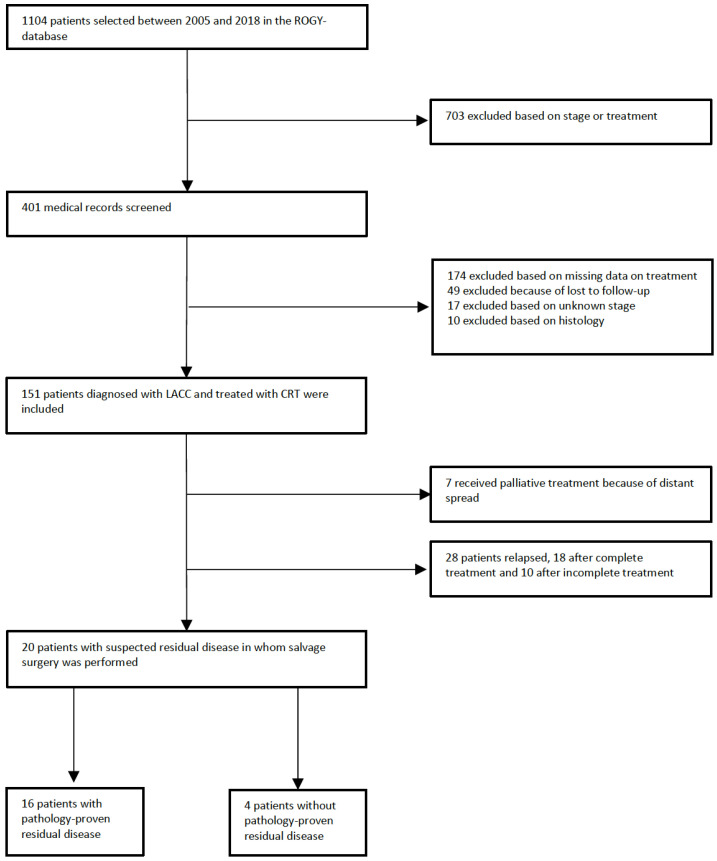
Patient selection flow chart. ROGY: Registration system Oncological Gynecology. LACC: locally advanced cervical cancer. CRT: chemoradiation therapy.

Between January 2005 and December 2018, 1104 patients with cervical cancer were identified in the ROGY database. Of these, 703 patients were excluded because they were diagnosed with early-stage cervical cancer and/or received a treatment other than CRT. Medical records of 401 patients with locally advanced cervical cancer were screened, and 174 patients were excluded because of missing data on treatment, 49 because of loss to follow-up, 17 because of unknown FIGO stage, and 10 because of different histological subtypes. In total, 151 patients met the inclusion criteria (Figure 1). 

### 3.1. Patient Characteristics 

A total of 117 patients (77%) received complete CRT treatment, and 34 patients received an incomplete CRT schedule. Of these thirty-four patients, fourteen patients did not receive brachytherapy because of their anatomy, twelve patients were treated with incomplete chemotherapy regimens because of medical reasons, four patients received neoadjuvant chemotherapy before CRT, three patients were treated with incomplete radiation therapy regimens, and one patient received lymphadenectomy before CRT. 

The median age at diagnosis was 51 years (range 27–85 years). Squamous cell carcinoma was diagnosed in 126 patients (83%) and adenocarcinoma in 21 patients (14%), while four patients were diagnosed with adenosquamous carcinoma (3%). After CRT, in total, 35 patients relapsed (23%), of whom 28 patients had locoregional recurrence and 7 patients received palliative treatment because of distant spread. This included 18 of the 117 fully treated patients (15%) and 10 of the 34 incompletely treated patients (30%) (Table 1). 

### 3.2. Salvage Surgery 

A total of twenty patients were treated with salvage surgery after CRT, of whom fourteen underwent a salvage hysterectomy and five salvage exenteration, while one patient was treated with a salvage lymphadenectomy and radiation therapy. The final decision to perform salvage surgery was made in one patient based on a positive biopsy, in one patient based on suspected locoregional residual disease on MRI, in four patients based on suspected locoregional residual disease on MRI and 18F[FDG]-PET/CT, in one patient based on a positive biopsy and MRI, and in two patients based on biopsy and 18F[FDG]-PET/CT, while one patient was operated at her own request and all other patients were operated based on positive biopsy, MRI and 18F[FDG]-PET/CT. 

A total of sixteen patients (80%) had pathology-proven locoregional residual disease on the salvage surgery specimen. Of the four patients without locoregional residual disease on surgery specimen, two were operated because suspected locoregional residual disease at MRI and 18F[FDG]-PET/CT without prior biopsy, one had suspected locoregional residual disease at MRI and 18F[FDG]-PET/CT with prior negative biopsy, and one was operated at her own request without suspected locoregional residual disease. A total of ten patients relapsed (50%) after salvage surgery, of whom six showed signs of local recurrent disease after salvage surgery (30%) and four showed signs of only distant metastases (20%). All patients who relapsed after salvage surgery were treated with palliative care (Figure 1). Among the patients with distant metastases, 50% did not receive brachytherapy, while all other patients with locoregional recurrences were adequately treated. Of the twenty patients who received salvage surgery, a total of six fistulas were diagnosed (three vesicovaginal, three rectovaginal), while five patients reported sexual problems after surgery and eleven patients reported having psychological symptoms after surgery. 

### 3.3. Diagnostic Procedures 

In total, 298 diagnostic MRI and 18F[FDG]-PET/CT were performed in 145 patients, of which 156 were MRI scans and 142 18F[FDG]-PET/CT. After CRT, 66 cervical biopsies were taken, with 18 of these showing locoregional residual disease (27%). Four patients received no radiological imaging. In these patients, the biopsy taken after CRT did not show locoregional residual disease. Only two patients received a CT scan during follow-up, and these scans did not show locoregional residual disease. In 78% of the patients, only one MRI was performed, and 12% of the patients had more than one MRI scan. Furthermore, 80% of the patients had one 18F[FDG]-PET/CT and 9% had more than one 18F[FDG]-PET/CT. The time from last CRT treatment until first MRI or 18F[FDG]-PET/CT was a median of 73 days (range 4–270 days). The time from last CRT treatment until biopsy or salvage surgery was a median of 111 days (range 47–378) and 180 days (range 47–675 days), respectively. The decision not to obtain histology was made at a median of 77 days (range 28–426 days) after the last CRT treatment (Table 2). The sensitivity and specificity for detection of locoregional residual disease by MRI in our study were 63.3% (CI 49.3–75.8) and 68.2% (CI 59.0–76.5), respectively. The NPV for MRI was 80.2% (CI 71.3–87.5) and the PPV was 47.7% (CI 35.8–59.7). The sensitivity and specificity for 18F[FDG]-PET/CT in our study were 63% (CI 48.7–76.0) and 76.2% (CI 66.9–83.8), respectively. The NPV for 18F[FDG]-PET/CT was 81.1% (CI 72.2–88.3), and the PPV was 55.8% (CI 42.2–68.7). The diagnostic accuracy of combining MRI and 18F[FDG]-PET/CT and both reporting same result was not significantly different, with sensitivity, specificity, NPV and PPV results of 67.9% (CI 49.5–83.1), 85.7% (CI 75.0–93.2), 84.2% (CI 73.3–92.1) and 70.4% (CI 51.8–85.2), respectively. No correlation was found between time after CRT and the reliability of radiological imaging in detecting locoregional residual disease (Table 3). 

## 4. Discussion

Our results show that the reliability of radiological imaging for the detection of locoregional residual disease after CRT in patients with locally advanced cervical cancer is limited. The diagnostic accuracies of individual MRI and 18F[FDG]-PET/CT or combined imaging do not show any significant differences. Therefore, routinely performing both MRI and 18F[FDG]-PET/CT at the same time for detection of locoregional residual disease after CRT can be omitted. MRI is the advised diagnostic procedure. Since the PPV of imaging is limited, a histological biopsy to confirm the presence of locoregional residual disease is advised prior to salvage surgery. No correlation was found between time after CRT and the reliability of radiological imaging in detecting locoregional residual disease. Based on our study, it is therefore, not possible to advise on the timing of the scan.

### 4.1. MRI

The accuracy of MRI depends on the time elapsed since the end of CRT. In the first six months after CRT, inflammation and edema contribute to a higher signal intensity on T2WI, which makes the diagnosis of locoregional residual disease more difficult [14]. The reported diagnostic accuracy of MRI and 18F[FDG]-PET/CT for locoregional residual disease detection in a meta-analysis from Sistani et al. is higher than our reported results. They reported sensitivity rates up to 73% and 86% for MRI and 18F[FDG]-PET/CT, respectively [12]. Discrepancies between the studies are possible due to the fact that the imaging results in our study were compared with immediate pathology-proven locoregional residual disease as well as locoregional recurrence during follow-up, while the study of Sistani et al. used pathology results in the treatment of patients with locally advanced cervical cancer as the ‘gold standard’. When locoregional microscopical residual disease was present, our scans would have missed the diagnosis of residual disease, but microscopical locoregional residual disease will eventually translate to locoregional recurrence. MRI, and 18F[FDG]-PET/CT cannot detect micro metastases, but these metastases may have a significant impact on prognosis and might affect treatment plans [15]. Also, we included patients from 2005 to 2018 to obtain longer follow-up periods, and in the last years, the availability and quality of radiological imaging improved, which may have led to a higher accuracy [16]. Since, both the literature and our study report limited diagnostic accuracy in the detection of locoregional residual disease using MRI, this diagnostic tool seems to be insufficient for predicting locoregional residual disease. However, based on a financial point of view and considering its availability and acceptability, it is the most appropriate imaging procedure. Therefore, during recent years, there has increasing attention towards standardizing treatment response after CRT, which could also increase the accuracy of MRI [17].

### 4.2. 18F[FDG]-PET/CT

The basis of 18F[FDG]-PET/CT imaging of tumors is the increased glucose metabolism in tumor tissue compared with normal tissue. Schwarz et al. showed that the metabolic response of 18F[FDG]-PET/CT can predict a long-term survival rate of 78% in patients with a complete metabolic response, 33% in patients with a partial metabolic response, and 0% in those with progressive disease [18]. The use of 18F[FDG]-PET/CT in patients with asymptomatic locoregional recurrence seems to lead to a higher overall survival [19], which indicates that 18F[FDG]-PET/CT is an accurate diagnostic technique for detecting locoregional residual disease before it becomes symptomatic. However, we were not able to show an association with time to CRT in our group of patients. In addition, Herrera et al. stated that 18F[FDG]-PET/CT plays an essential role in evaluating lymph nodal status and distant metastases in patients with cervical cancer [20]. Therefore, 18F[FDG]-PET/CT can play a significant role in the follow-up period, but 18F[FDG]-PET/CT is not a sufficient diagnostic procedure to predict locoregional residual disease. Currently, there are new tracers in PET/CT diagnostics. The Fibroblast Activation Protein Inhibitor (FAPI) PET detects the fibroblast activation protein (FAP), which is overexpressed by cancer-associated fibroblasts (CAFs) in the cancer microenvironment. The 68Ga-FAPI-PET/CT technique shows promising results in detecting primary cervical cancer and in differentiating between metastatic and reactive lymph nodes. 68Ga-FAPI-PET/CT may be more accurate than 18F[FDG]-PET/CT in detecting residual cervical cancer after chemoradiation [21,22,23]. 

### 4.3. Other Diagnostic Procedures 

In some hospitals, standard biopsy after CRT is part of the protocol. Determination of locoregional residual disease by a cervical biopsy after CRT has previously shown a sensitivity of 89% and a specificity of 100%, indicating that confirmation of the presence of locoregional residual disease by a biopsy seems to be advisable before performing salvage surgery when locoregional residual disease is suspected [24]. In addition to MRI and 18F[FDG]-PET/CT, there are other diagnostic procedures which seem valuable for detection of locoregional residual disease. PET/MRI shows promising results in detecting locoregional residual disease. In a pilot study, Mongula et al. showed an increased diagnostic confidence in evaluating treatment response in 80–90% of all patients [17]. Sawicki et al. reported a sensitivity of 100% of PET/MRI in 71 women with suspected pelvic recurrence (cervical, ovarian, endometrial, vulvar, and vaginal) cancer detection. These patients had undergone surgery and/or CRT after initial diagnosis and had been considered cancer-free for ≥six months [25]. In the future, these promising results may contribute to detection of locoregional residual disease. However, PET/MRI is not used in all hospitals due to limited availability and high costs [26]. In addition to radiological imaging, our study indirectly evaluates the performance of the multidisciplinary team. In this multidisciplinary meeting, the results of the imaging are discussed and a joint conclusion is formed.

### 4.4. Salvage Surgery

In our study, four out of twenty patients did not show locoregional residual disease in the salvage surgery specimen. In two of these cases, the biopsy did not show locoregional residual disease prior to surgery; therefore, these patients were unnecessarily exposed to the risks of salvage surgery. The timing of salvage surgery, as well as the timing of radiological imaging, remains important. The EMBRACE-1 study showed an overall complete remission (CR) of CRT of 98%. At three-month follow-up, 81 out of 1318 patients were observed with persistent disease. Of these, 21 patients had true persistent disease evolving into progressive disease, while 60 patients achieved CR at six- to nine-month follow-up without intervening treatment [27]. This indicates that it takes some time to reach complete response due to the enduring effects of CRT. This study was published after we included the patients in our study, but based on these results, we have possibly drawn conclusions too early in our study. 

Even after salvage surgery, there is still a high reported locoregional recurrence rate. We reported an overall relapse rate of 50%, of which 30% was diagnosed with locoregional recurrence and 20% with distant failure. The recurrence rate after salvage surgery in our study is based on small numbers and is comparable with the reported literature. Two previously published systematic reviews reported overall relapse rates of 32% and 31% after salvage surgery [10,28]. In addition to the recurrence rate, Sardain et al. reported that salvage exenteration for recurrent disease increased the five-year OS from 20% to 64% [29]. Based on these results, it is important to counsel patients about the survival benefits and disadvantages before treatment with salvage surgery. Therefore, further investigations on the timing of radiological imaging to detect locoregional residual disease as well as the timing of salvage surgery are important.

### 4.5. Study Limitations

One limitation of this study is its retrospective nature. The data collection relies on the quality of data in the medical records. Patients were included from 2005 onwards, which makes data collection more difficult due to the absence of electronic patient files. This resulted in a high percentage of patients excluded based on missing data. Another limitation arising from this inclusion period is the improvements in the quality of imaging techniques and CRT in recent years. We used the 18F[FDG]PET/CT technique in our study. Recent research shows promising results for the 68Ga-FAPI-PET/CT technique, which could lead to higher diagnostic accuracy for PET-CT. Imaging for this specific question should be standardized in performance and assessment. In our study, different reference standards were used for verification because pathology results were not available from all patients. Therefore, verification bias needs to be considered. Therefore, the low accuracy from radiological imaging could be caused by the reference test we used, namely, locoregional recurrence at any point during follow-up, resulting in a lower diagnostic accuracy. Statistical uncertainty must be considered based on the low number of patients and wide confidence intervals. The results of our study are useful for western countries, based on the generalizability of cervical cancer diagnosis and treatment.

### 4.6. Implications for Practice

Based on the results of our study, combining MRI and 18F[FDG]-PET/CT does not improve predictive values. Routine use of both MRI and 18F[FDG]-PET/CT in follow-up after CRT should, therefore, be avoided. When imaging is routinely combined, patients are unnecessarily exposed to multiple scans. Therefore, we recommend performing only an MRI scan during follow-up based on its availability, acceptability, and healthcare costs. In addition, 18F[FDG]-PET/CT should be performed prior to salvage surgery to rule out distant metastases and can be performed when there is any doubt about a diagnosis. If locoregional residual disease is suspected on MRI, histological confirmation of locoregional residual disease and 18F[FDG]-PET/CT are warranted to rule out distant metastasis before performing salvage surgery. The optimal timing to perform radiological imaging could not be determined. Therefore, timing to perform diagnostic procedures after CRT should be further investigated. 

## 5. Conclusions

Salvage surgery is a procedure with a high morbidity. Therefore, critical selection of patients with true locoregional residual disease is important. The reliability of MRI and 18F[FDG]-PET/CT is limited, and combining both techniques to rule out locoregional residual disease does not show significant improvement in reliability. Therefore, we recommend performing only MRI during follow-up based on its availability, acceptability, and healthcare cost. To prevent over-treatment by salvage surgery, we advise to confirm the presence of locoregional residual disease by a biopsy and to perform 18F[FDG]-PET/CT to rule out distant metastases before performing salvage surgery. 

## Figures and Tables

**Table 1:** Patient characteristics.

		n (%)
Total		151
Treatment	Complete	117 (77%)
	Incomplete	34 (23%)
Histology	Squamous cell carcinoma	126 (83%)
	Adenocarcinoma	21 (14%)
	Adenosquamous cell carcinoma	4 (3%)
FIGO stage 2009	IB1	14 (9%)
	IB2	15 (10%)
	IIA1	5 (3%)
	IIA2	7 (5%)
	IIB	69 (46%)
	IIIA	4 (3%)
	IIIB	27 (18%)
	IVA	10 (7%)
Salvage surgery	Salvage hysterectomy	14
	Salvage exenteration	5
	Lymphadenectomy + RT	1
Recurrence	After CRT	28
	After salvage surgery	10

CRT: chemoradiation therapy. FIGO: Federation of Gynecology and Obstetrics.

**Table 2:** Information about imaging and the number of scans performed per patient during follow-up.

Information about Imaging	n (%)
Number of patients with scans in follow-up	145
Number of patients without MRI or 18F[FDG]-PET/CT scan in follow-up	6
Number of scans performed in total	299
Number of MRI	156
Number of 18F[FDG]-PET/CT	143
Number of scans per patient	
MRI *	
0	22
1	113 (78%)
2	11 (8%)
3	3 (2%)
4	0 (0%)
5	1 (1%)
6	0 (0%)
7	1 (1%)
18F[FDG]-PET/CT *	
0	22
1	116 (80%)
2	12 (8%)
3	1 (1%)
Patients received only MRI	16 (11%) *
Patients received only 18F[FDG]-PET/CT	16 (11%) *
Patients received 1 MRI and 1 18F[FDG]-PET/CT	93 (62%) *
Patients received at least 1 MRI and 1 18F[FDG]-PET/CT	20 (13%) *
Time from CRT until first scan, median (range)	
First MRI	73 days (4–232 days)
First 18F[FDG]-PET/CT	75 days (28–494 days)
First scan MRI or 18F[FDG]-PET/CT	73 days (4–270 days)
Time from last CRT treatment until obtained pathology, median (range)	
Time until biopsy	111 days (47–378 days)
Time until salvage surgery	180 days (47–675 days)
Time from last CRT until the decision that pathology was not necessary, median (range)	77 days (28–426 days)

* Percentages are calculated based on number of patients, n = 145.

**Table 3:** Diagnostic accuracy of MRI and 18F[FDG]-PET/CT scans for detection of locoregional residual disease.

	Sensitivity (95% CI)	Specificity (95% CI)	NPV (95% CI)	PPV (95% CI)	Number of Scans
MRI					
Overall	63.3% (49.3–75.8)	68.2% (59.0–76.5)	80.2% (71.3–87.5)	47.7% (35.8–59.7)	156
<12 weeks	73.9% (75.3–94.6)	70.2% (54.1–88.7)	87.0% (75.3–94.6)	50.0% (33.7–66.3)	80
12–18 weeks	42.9% (19.8–68.3)	71.4% (55.3–84.5)	75.8% (59.6–88.1)	37.5% (61.8–96.0)	49
>18 weeks	66.7% (38.7–88.2)	53.3% (29.1–76.5)	66.7% (38.7–88.2)	53.3% (29.1–76.5)	27
18F[FDG]-PET/CT					
Overall	63.0% (48.7–76.0)	76.2% (66.9–83.8)	81.1% (72.2–88.3)	55.8% (42.2–68.7)	143
<12 weeks	57.9% (35.8–78.0)	81.5% (69.8–90.3)	84.6% (73.3–92.7)	52.4% (31.7–72.5)	74
12–18 weeks	53.8% (27.9–78.4)	76.7% (59.7–89.2)	79.3% (62.5–91.2)	50.5% (25.5–74.5)	43
>18 weeks	76.9% (50.5–93.7)	53.8% (27.9–78.4)	70.0% (39.3–91.5)	62.5% (38.2–83.0)	26
First MRI	60.5% (44.7–75.0)	76.1% (66.7–84.0)	82.4% (73.3–89.4)	51.1% (36.8–65.3)	113
First 18F[FDG]-PET/CT	57.9% (42.1–72.7)	80.2% (71.3–87.5)	82.0% (73.2–89.0)	55.0% (39.6–69.7)	116
Last MRI	100%	30.0% (8.5–60.7)	100%	46.2% (21.6–72.1)	16
Last 18F[FDG]-PET/CT	85.7% (50.6–99.1)	16.7% (10.0–55.4)	50.0% (3.8–80.6)	54.5% (26.5–80.6)	13
Combination MRI and 18F[FDG]-PET/CT, both reporting same result *	67.9% (49.5–83.1)	85.7% (75.0–93.2)	84.2% (73.3–92.1)	70.4% (51.8–85.2)	84
MRI and 18F[FDG]-PET/CT, at least one reporting suspected residual disease *	76.9% (62.2–88.2)	59.3% (48.4–69.5)	84.2% (73.3–92.1)	47.6% (35.6–59.9)	120

* MRI and 18F[FDG]-PET/CT were performed within four weeks of each other. NPV: negative predictive value. PPV: positive predictive value.

## Data Availability

No new data were created or analyzed in this study. Data sharing is not applicable to this article.
